# Immunogenicity of the CoronaVac vaccine in children: a real-world study

**DOI:** 10.3389/fimmu.2024.1504935

**Published:** 2024-12-23

**Authors:** Wbeimar Aguilar-Jimenez, Ana Lucia Rodriguez-Perea, Mateo Chvatal-Medina, Paula A. Velilla, Wildeman Zapata-Builes, Laura M. Monsalve-Escudero, Maria I. Zapata-Cardona, Jorge Humberto Tabares-Guevara, Daniel S. Rincón, Juan C. Hernandez, Yulied Tabares, Liliana Lopez-Carvajal, Maria T. Rugeles

**Affiliations:** ^1^ Grupo Inmunovirología, Facultad de Medicina, Universidad de Antioquia UdeA, Medellín, Colombia; ^2^ Grupo Infettare, Universidad Cooperativa de Colombia, Medellín, Colombia; ^3^ Grupo de Investigación Clínica PECET (GIC-PECET), Universidad de Antioquia, Medellín, Colombia

**Keywords:** real-world study, COVID-19 vaccine, children, immunogenicity, neutralizing antibodies, omicron variant, CoronaVac, cellular response

## Abstract

**Background:**

Despite its proven effectiveness and safety, there are limited real-world data on CoronaVac’s immunogenicity in children, especially in lower-income countries, particularly for SARS-CoV-2 variants. We present a real-world study evaluating CoronaVac’s immunogenicity in Colombian children stratified by previous exposure to this virus.

**Methods:**

89 children aged 3-11 years were enrolled (50 Non-Exposed and 39 Exposed). Saliva samples were collected every 15 days to monitor potential SARS-CoV-2 infection, and blood samples were taken at two and six months after vaccination, to evaluate immunogenicity. Total IgG and IgA antibodies were measured by ELISA, and neutralizing titers against B.1, Delta, Mu, and Omicron variants were assessed by plaque reduction assay. T-cells were stimulated with wild-type and Omicron peptide pools to analyze activation-induced markers, memory phenotype, cytotoxic molecules, and cytokine production by flow cytometry.

**Findings:**

CoronaVac was well tolerated, with only 7.8% infection incidence in both Exposed and Non-Exposed groups. It elicits a robust humoral response through IgG, IgA, and neutralizing antibodies against all variants. Despite waning, most participants maintained neutralizing titers ≥20 over time. CoronaVac also induced a polyfunctional cellular response against various strains, albeit reduced against Omicron, regardless of prior exposure. This response, characterized by IFN-γ/TNF-α and cytotoxic molecule production, was more pronounced in CD4^+^ than in CD8^+^ T-cells and remained detectable even after 6 months.

**Interpretation:**

CoronaVac induces robust humoral and cellular immune responses against various variants in children, suggesting cross-recognition. However, these responses diminish over time, particularly in the context of variants, indicating the need for booster doses.

## 1 Introduction

Despite the competence of the adaptive immune response against SARS-CoV-2, residual memory after natural infection is often insufficient to prevent reinfection. Vaccines have been crucial for mitigating morbidity and mortality around the globe but are susceptible to the same problem, due to the emergence of variants with critical antigenic drift (1). Consequently, continued efforts have been made to evaluate the effectiveness and safety of the available vaccines, mainly focusing on the study of humoral immune responses. Although less studied, cellular immunity appears to be a protagonist, as compelling evidence has pointed to the importance of cross-reactivity and preserved response to novel variants even with waning antibodies (2).

Over 3 million Colombian children have received at least one dose of CoronaVac (Sinovac) (2). Initially, Colombian children from 3-11 years old were vaccinated exclusively with CoronaVac since national regulatory guidelines only allowed the administration of this vaccine to this age group, which also created a unique opportunity to evaluate the immunogenicity under real life conditions (3). However, despite impressive public health efforts, further studies evaluating immune response kinetics and immunogenicity in real-world scenarios in vaccinated children are needed. Undoubtedly, it is necessary to assess its impact on protection against disease and to evaluate the waning of immunity in this population.

Although valuable evidence has surfaced, most of it comes from phase 1-3 studies, which often lack factors representing real-world conditions, particularly in children who are underrepresented in research. This is particularly true in Latin America, where socioeconomic inequalities deepen the necessity for such studies.

Notably, CoronaVac-vaccinated children from Chile showed robust CD4+ responses against structural proteins (S, N, and M), and remarkable AIM+ T-cell responses against Omicron (4). However, this phase 3 study lacked polyfunctional characterization of cellular responses as well as differential responses to specific SARS-CoV-2 variants or effects of hybrid immunity.

Consequently, we designed this study to describe and determine the characteristics of adaptive immune responses against SARS-CoV-2 in children aged 3-11 years old, comparing those exclusively immunized with CoronaVac to those with hybrid immunity due to exposure or infection with SARS-CoV-2, and further boosting with CoronaVac, seeking to provide evidence on the vaccine’s neutralizing response and cellular response against several variants of SARS-CoV-2.

## 2 Materials and methods

### 2.1 Study design

A prospective longitudinal cohort study was conducted in Medellín, Colombia (June 2022 to July 2023). It involved fully vaccinated children aged 3-11 years who received two doses (0.5mL ~600SU each) of CoronaVac^®^ vaccine (Sinovac Life Sciences), with an interdose interval of 28-100 days. The study was semi-blinded, with analysts unaware of exposure status until sample processing was completed.

The children were classified into two groups: “Exposed” and “Non-Exposed.” The “Exposed” group included children who met at least one of the following criteria before vaccination: (1) documented SARS-CoV-2 infection confirmed by antigen or RT-PCR testing, or (2) a reported history of close contact with a household member who tested positive for COVID-19 within 14 days of the household member’s diagnosis. The “Non-Exposed” group included children who did not meet either criterion.

**Figure 1:**
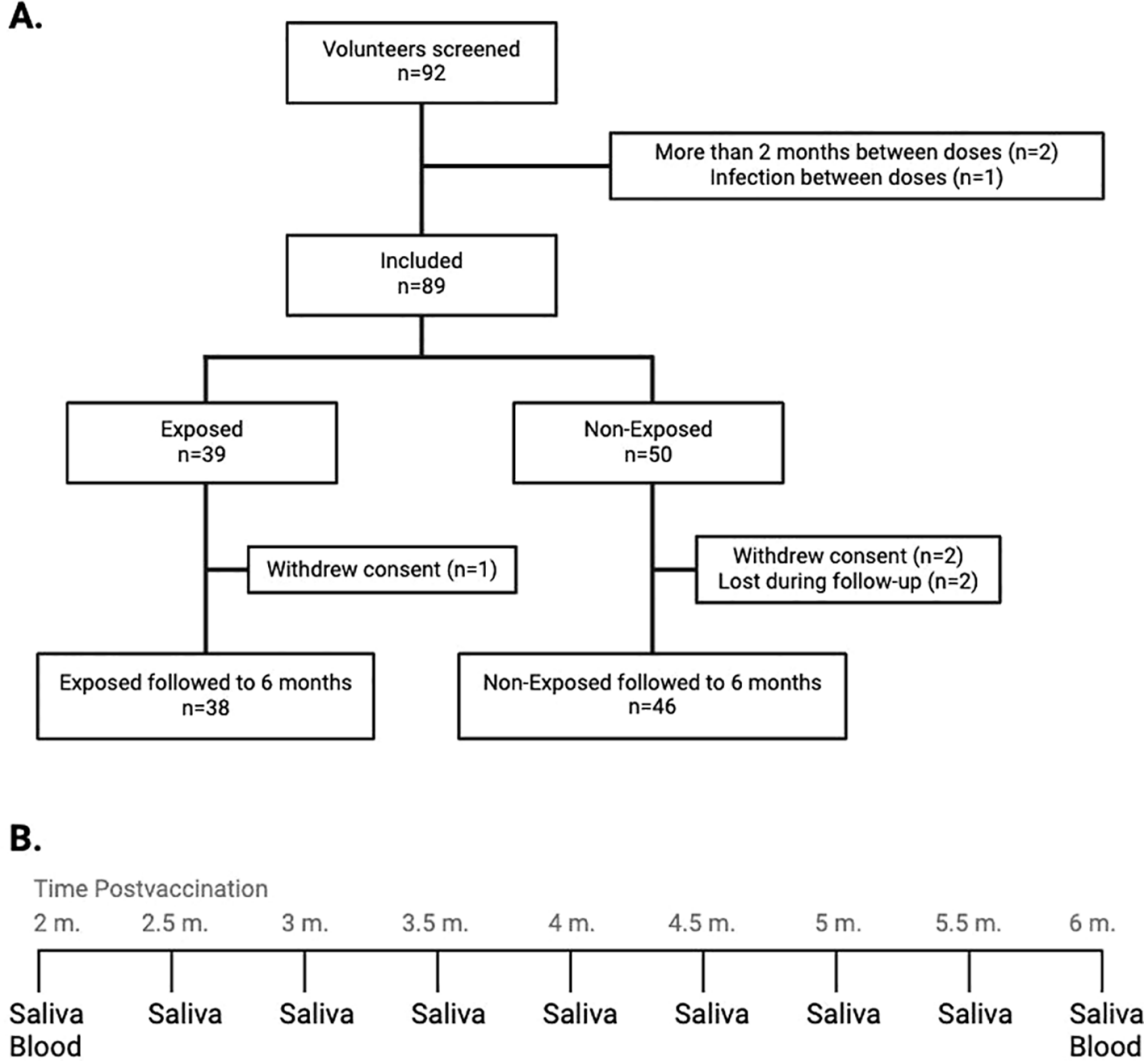
**(A)** Flow diagram representing the inclusion and follow-up of study participants: The graph describes the number of screened volunteers, exclusion before assignment into groups according to their exposure status, and the final distribution of the included children at the end of the follow-up. **(B)** Saliva and blood collection at the 9 time points throughout the follow-up. From the initial samples of both saliva and blood, saliva samples were withdrawn every 15 days for monitoring for SARS-CoV-2 infection, up to the final withdrawal at six months post-vaccination, when both saliva and blood samples were taken. m, months.

Saliva was collected with DNA/RNA shield (Zymo, USA) every 15 days for SARS-CoV-2 testing via RT−qPCR, and blood was drawn at 2- and 6-months post-vaccination for immune response evaluation (**Figure 1**). Data related to the safety of the vaccine were also recorded. The reagents used are detailed in **Supplementary Table S1**. The study adhered to Helsinki Declaration and Resolution 8430 of 1993 of the Minister of Health of Colombia (mainly Chapter III “Research on minors”). It was approved by the Ethics Committee of the Universidad de Antioquia (Act 027/2022).

### 2.2 Detection of the SARS-CoV-2 viral genome by RT−qPCR

The RNA was extracted from saliva samples using a SaMag viral nucleic acid extraction kit and a SaMag-12 nucleic acid extractor (Sacace Biotechnologies, IT). RT−qPCR was performed using a BGI 2019-nCoV RT−PCR kit on a CFX-96 thermal cycler (Bio-Rad, USA).

### 2.3 Plasma and peripheral blood mononuclear cell collection

Plasma was obtained by centrifuging blood at 800 × g for 10 minutes and stored at -80°C. Peripheral blood mononuclear cells (PBMCs) were isolated using Ficoll-Histopaque centrifugation gradient.

### 2.4 Quantification of antibodies against S and N SARS-CoV-2 proteins by ELISA

Anti-S IgG and anti-S/anti-N IgA antibodies against SARS-CoV-2 (Wuhan P0DTC2 and YP_009724397) were detected in plasma by ELISA (MyBioSource. MBS7612298/MBS7612290) following the manufacturers’ recommendations.

### 2.5 Viruses

Colombian SARS-CoV-2 isolates were used for neutralization assays: An ancestral WT lineage (B.1, EPI_ISL_536399), Delta (B.1.617.2, EPI_ISL_5103929), Mu (B.1.621, EPI_ISL_4005445), and Omicron (BA.1.1, EPI_ISL_8374770). Virus manipulation was conducted in a biosafety level-3 laboratory.

### 2.6 Plaque reduction neutralizing antibody assays

Neutralizing antibody titers from plasma were assessed via a plaque reduction neutralization test (PRNT) as previously described (5), using serial dilutions (1:20 to 1:5120) of heat-inactivated plasma mixed with 200 PFU/0.1 mL of each virus, and incubated with Vero E6 cells (ATCC CRL-1586). For Omicron, 15 µg/mL of trypsin-EDTA was added before incubation. After 4-5 days, plaques were counted (**Supplementary Figure S1**), and titers were measured as the dilution required to neutralize 50% of the viral plaque formation (PRNT50). A titer of ≥20 was considered positive for SARS-CoV-2 neutralizing antibodies (6).

### 2.7 SARS-CoV-2 peptides

Pools of lyophilized SARS-CoV-2 peptides (>70% purity by HPLC and MS, from GenScript, China) derived from WT and Omicron were designed based on literature and peptide affinity to human leukocyte antigens (HLAs) prevalent in Colombia (7) using TepiTool (http://tools.iedb.org/tepitool/). Each pool (WT and Omicron) consisted of 117 peptides (8–22 aa in length), 52 lineage-specific, and 65 conserved, from the spike (S), envelope (E) membrane (M) and nucleocapsid (N) proteins (**Supplementary Table S2**). The peptides were reconstituted at 2000 µg/mL in DMSO.

### 2.8 T-cell stimulation cultures

Thawed PBMCs from 34 children (17 per group) were cultured in 96-well V-bottom plates at 8x10^5^ cells/mL in RPMI-1640 medium with 10% FBS, stimulated with 1 µg/mL anti-CD28 and anti-CD49d antibodies along with 5 µg/mL peptides from WT or Omicron for 18 hours at 37°C. Unstimulated PBMCs with the same antibodies and DMSO concentrations as the peptides served as negative controls (background). PBMCs stimulated with 10 µg/mL phytohemagglutinin or 40 ng/mL PMA and 500 ng/mL ionomycin were used as positive controls. All conditions included anti-human CD107a.

### 2.9 Activation-induced marker assay

The PBMCs were stimulated as described above, labeled with the antibodies for AIM at 4°C for 30 minutes, acquired on an LSR Fortessa flow cytometer (Becton Dickinson, BD) and analyzed using FlowJo Software V10.9.0 (Tree Star, Inc., Ashland, USA). AIM^+^ CD4^+^ T-cells (CD137^+^OX40^+^ and OX40^+^CD25^+^) and AIM^+^ CD8^+^ T-cells (CD69^+^CD137^+^ and CD25^+^CD107^+^) were detected. The data were reported after background subtraction. Responder’s percentage was set at 0.02% for CD4^+^ and CD8^+^ T-cells. The differentiation state of AIM^+^ T-cells was evaluated by examining central, effector memory, and terminally differentiated (TEMRA) cell subpopulations.

### 2.10 Intracellular cytokine staining

After PBMCs stimulation as before, this time adding 10 µg/mL brefeldin A/monensin, cells were labeled with Viability Dye, anti-CD3, anti-CD4, and anti-CD8 at 4°C for 30 minutes. Then, cells were fixed and permeabilized using FoxP3/transcription factor staining buffer and labeled with antibodies against IFN-γ, TNF-α, IL-2, IL-10, perforin, and granzyme B at 4°C for 30 minutes. At least 100,000 events were acquired on an LSR Fortessa cytometer (BD) and analyzed using FlowJo V10.9.0 (Tree Star). Compensation for fluorochrome spillover was achieved using unstained and single-stained cells with each antibody.

Data was reported after background subtraction. Polyfunctional T-cells were evaluated using Boolean analysis and SPICE software v6.1 (Vaccine Research Center, NIAID/NIH, USA). Responders were defined as individuals with a value >0.001 after background subtraction. The polyfunctional index was obtained using the Funky Cells Toolbox, version 0.1.0 beta (http://www.funkycells.com/main).

### 2.11 Cytokine measurement by cytometric bead array

The CBA Enhanced Sensitivity Flex Set system (BD) was used to detect IL-6, IL-10, IL-2, IL-4, and IL-17a levels in supernatants of PBMCs stimulated with SARS-CoV-2 peptides after background subtraction, according to the manufacturer’s instructions. Fluorescence was determined using flow cytometry (CitoFLEX, Beckman Coulter), and cytokine levels were analyzed using FlowJo V10.9.0 (Tree Star).

### 2.12 Statistical analysis

Since the data were not normally distributed according to the Shapiro-Wilk test, nonparametric tests were used for analysis, except for total specific anti-SARS-CoV-2 IgG and IgA, which were analyzed using paired T-tests. The Wilcoxon test estimated differences over time for each variant. The Mann-Whitney test compared Non-Exposed and Exposed at the same time point. The Wilcoxon or Friedman/Dunn correction tests compared variants/lineages for each group. For neutralizing antibodies, the geometric mean titer (GMT) and fold-change of the variants compared to the ancestral lineage were described. Statistical analysis and graphics were generated using GraphPad Prism 10.0.

## 3 Results

### 3.1 Sociodemographic characteristics and incidence of infection

Out of the 92 screened volunteers, 89 initiated the four-month follow-up. There were no differences in sex or age between Exposed and Non-Exposed children. Among the 7 children who tested positive for SARS-CoV-2 during the follow-up, 3 had a history or epidemiological link to SARS-CoV-19 infection, and 2 reported mild symptoms, with no differences between the Exposed and Non-Exposed groups (**Table 1**). A total of 84 children (94.4%) completed the follow-up to evaluate the immunogenicity of CoronaVac^®^; 38 were allocated to the “Exposed” and 46 to the “Non-Exposed” group. Reasons for withdrawal are shown in **Figure 1**.

**Table 1:** Age and sex distribution of participants.

		History of COVID-19 before vaccination	
		Non-Exposed n=50	Exposed n=39
Sex	Female (%)	34 (68)	21 (53.8)
	Male (%)	16 (32)	18 (46.2)
Age (years)	Median (IQR)	3 (3 – 6)	4 (3 – 7)
Positivity by PCR during follow-up	Number (%)	4 (8.0%)	3 (7.7%)

IQR, Interquartile range.

### 3.2 Safety and reactogenicity

The predominant adverse event post-vaccination was pain at the injection site (30%), and the most frequent systemic adverse event was fever following both doses. There were no significant differences in the evaluated effects between doses or exposure status, but “other” effects showed a significant association (related to respiratory symptoms) with exposure status (**Table 2**).

**Table 2:** Relationship of local and systemic vaccine adverse events.

Adverse Event		First dose		Second dose		p^1^ value *	p^1^ value **
		Non-Exposed n=50 (%)	Exposed n=39 (%)	Non-Exposed n=50 (%)	Exposed n=39 (%)		
**Local**	Local warmth	7 (14)	6 (15.4)	3 (6)	5 (12.8)	0.26	0.37
	Erythema	5 (10)	5 (12.8)	3 (6)	5 (12.8)	0.26	0.26
	Swelling	5 (10)	1 (2.6)	3 (6)	2 (5.1)	0.86	0.24
	Local pain	19 (38)	12 (30.8)	15 (30)	10 (25.6)	0.65	0.88
**Systemic**	Fever	10 (20)	4 (10.3)	3 (6)	6 (15.4)	0.15	0.96
	Fatigue/malaise	6 (12)	1 (2.6)	4 (8)	2 (5.1)	0.59	0.10
	Headache	4 (8)	2 (5.1)	2 (4)	1 (2.6)	0.71	0.50
	Shivering	2 (4)	0 (0)	0 (0)	0 (0)	NA	NA
	Diarrhea	0 (0)	0 (0)	0 (0)	1 (2.6)	NA	NA
	Vomiting	1 (2)	1 (2.6)	0 (0)	0 (0)	NA	NA
	Abdominal pain	0 (0)	0 (0)	0 (0)	1 (2.6)	NA	NA
	Myalgias/Arthralgias	3 (6)	3 (7.7)	0 (0)	2 (5.1)	NA	NA
	Rash	0 (0)	1 (2.6)	0 (0)	0 (0)	NA	NA
	Other: cough, nasal congestion and runny nose	1 (2)	5 (12.8)	1 (2)	4 (10.3)	0.09	0.007

^1^Chi-squared test. NA, Non-applicable, some cells are empty. *Difference between vaccine doses.

**Difference between groups (No Exposed vs. Exposed). p-value >0.05 is considered significant.

### 3.3 CoronaVac^®^ induces a robust humoral response with neutralizing capacity against SARS-CoV-2 variants

**Figure 2:**
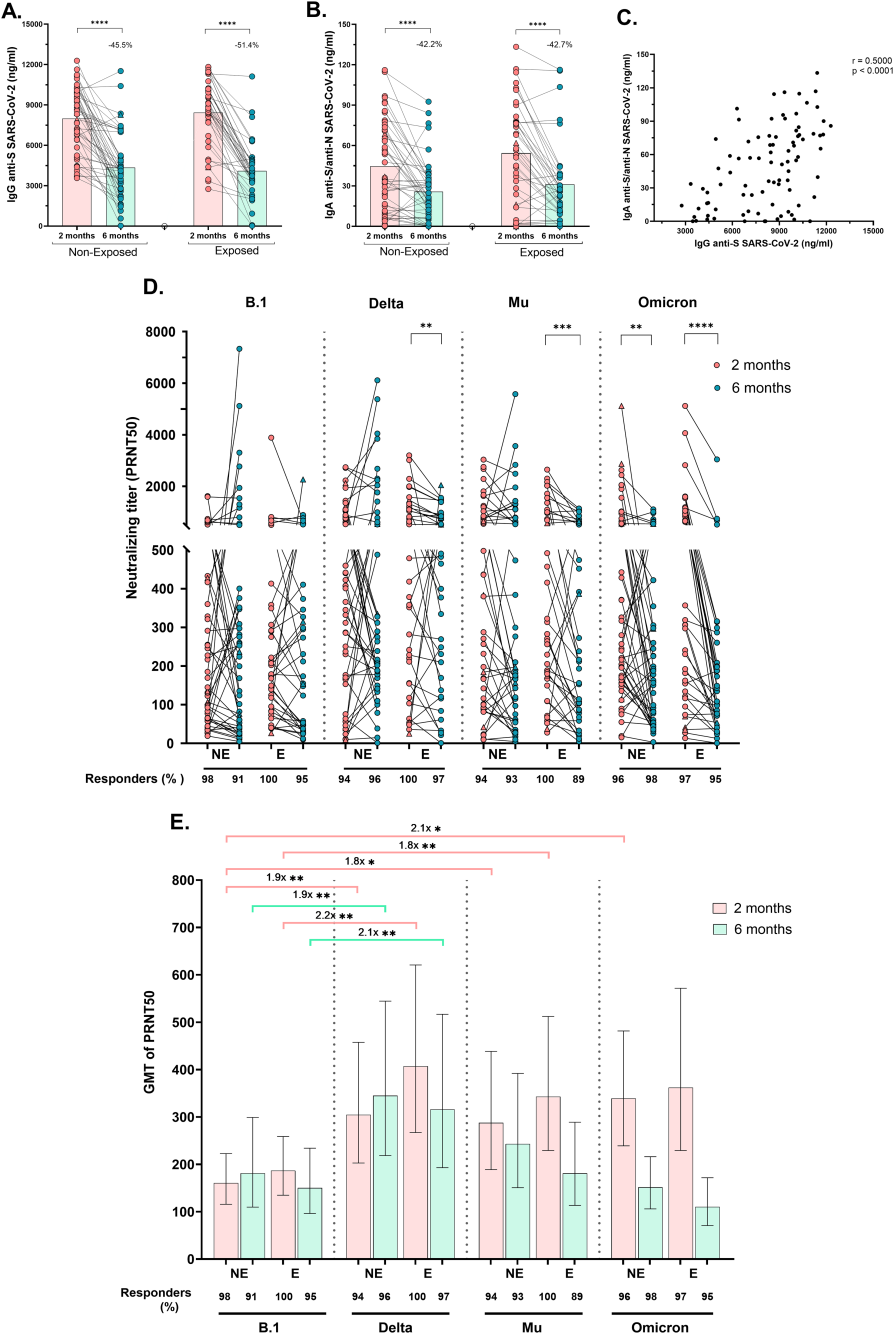
Specific IgG, IgA and neutralizing antibody titers after receiving a complete vaccine scheme according to SARS-CoV-2 exposure: Antibody titers were quantified by ELISA and reported as ng/mL. Anti-S (WT strain) IgG antibodies titers **(A)** and Anti-S/anti-N (WT strain) IgA antibodies titers **(B)** in Non-Exposed and Exposed subjects. Pearson correlation analysis of IgG anti-S SARS-CoV-2 titers vs IgA anti-S/anti-N SARS-CoV-2 titers (r = 0.5000, p<0.0001) **(C)**. Neutralizing titers (PRNT50) of CoronaVac-vaccinated individuals against an ancestral lineage (D614G strain) and three SARS-CoV-2 variants (Delta, Mu, and Omicron) determined via a 50% plaque reduction neutralization test are shown. The figures show the results at two- and six-months post-vaccination in 46 Non-Exposed (NE) and 38 Exposed **(E)** subjects. Statistical comparisons for **(A, B)** were conducted using a paired T-test, while for **(D)**, a Wilcoxon test was employed. In **(E)** the bars represent the geometric mean titer (GMT) of the PRNT50s, along with their 95% confidence intervals (CIs). The fold change in GMTs among variants/lineages is depicted. The Friedman and Dunn tests were employed for multiple comparisons to compare the neutralizing titers among the variants for each exposure group and time point. Seven children who tested positive for SARS-CoV-2 by RT-PCR during this study are represented by triangles in the figures. **(D, E)** display the frequency of responders, representing individuals with antibody titers >20. *p<0.05, **p<0.01, ***p<0.001, ****p<0.0001.

Circulating SARS-CoV-2-specific IgG (anti-S) and IgA (anti-S/anti-N) antibodies were measured. All children exhibited substantial levels of specific IgG and IgA, 2 months post-vaccination, which significantly decreased at 6 months post-vaccination, by at least 45.5% for IgG and by 42.2% for IgA, regardless of SARS-CoV-2 exposure (p<0.0001, **Figures 2A, B**). Furthermore, the IgG and IgA titers were positively correlated (r=0.50, p<0.0001). **Figure 2C**).

Neutralizing activity (PRNT50 titers ≥20) against B.1 and three variants was observed in >96% of subjects two months post-vaccination and remained at over 91.7% at six months post-vaccination (**Figures 2D, E**). The PRNT50 GMT against Delta and Mu (after two and six months), and against Omicron (at two months) was greater than against B.1 in both Exposed and Non-Exposed subjects, with most differences being statistically significant (**Figure 2E**). Although the median PRNT50 titers against the ancestral strain remained stable over time in both groups, significantly lower PRNT50 titers against Delta (p=0.004), Mu (p=0.0003), and Omicron (p<0.0001) were detected in the Exposed group at six months post-vaccination compared to two months. In Non-Exposed children, the reduction in neutralizing titers over time was observed only against Omicron (**Figure 2D**, p=0.0034).

### 3.4 CoronaVac^®^ induces AIM+ and cytokine-producing WT- and omicron-specific CD4^+^ and CD8^+^ T-cells

**Figure 3:**
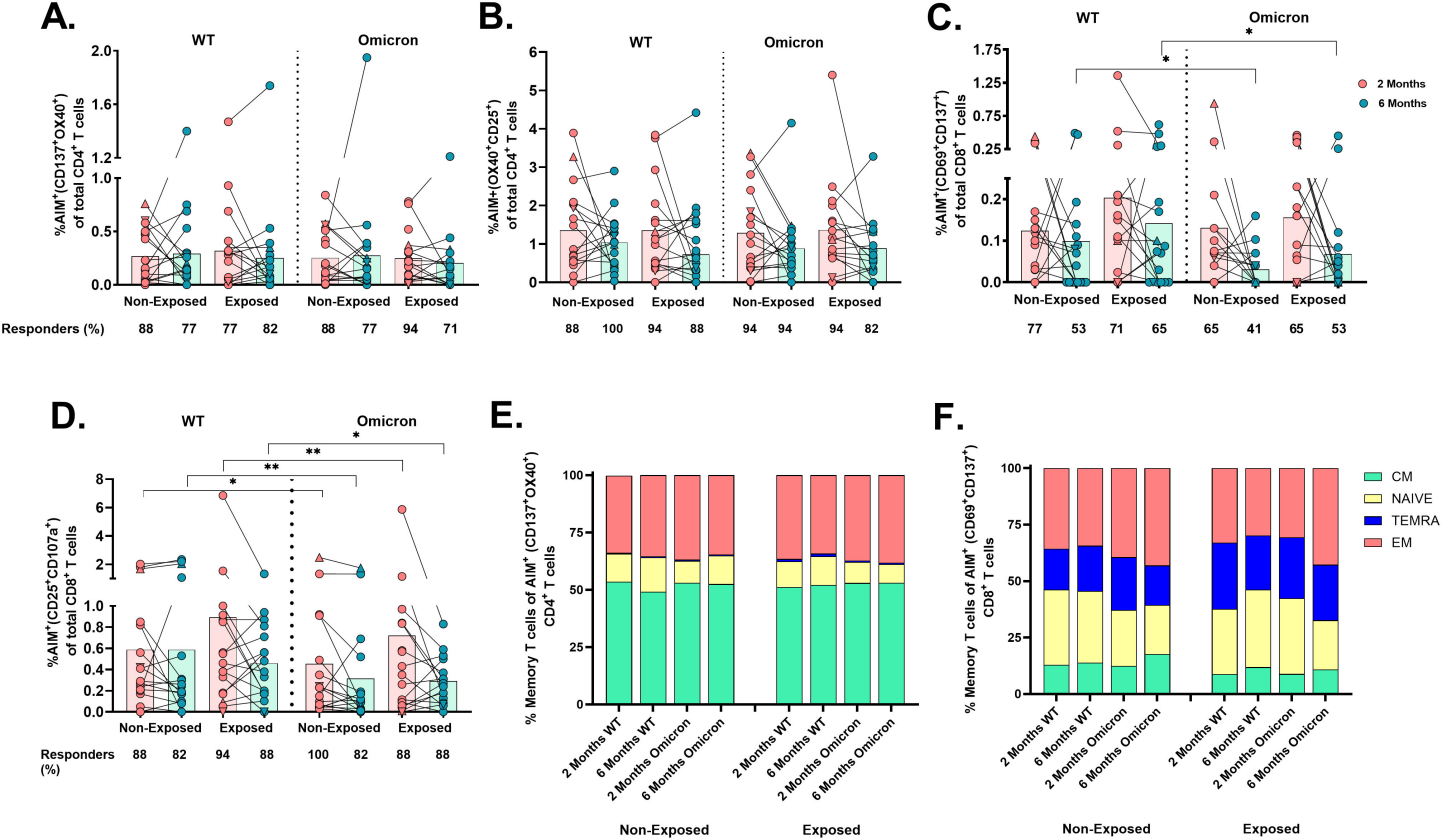
Frequency of AIM^+^OX40^+^ cells and memory profile after two doses of the CoronaVac vaccine: The percentages of AIM+CD4+ T-cells (CD137+OX40+) **(A)**, AIM+CD4+ T-cells (OX40^+^CD25^+^) **(B)**, AIM^+^CD8^+^ T-cells (CD69^+^CD137^+^) **(C)**, and AIM^+^CD8^+^ T-cells (CD25^+^CD107a^+^) **(D)** in response to peptide pools from the WT strain and the Omicron variant are shown (n=17 per group), gated from CD4^+^ T and CD8^+^ T-cells, respectively. AIM^+^ T-cells were determined after subtracting the values obtained for the negative control, and individual values for AIM^+^ **(A–D)** are depicted. In the figures, four out of seven children who tested positive for SARS-CoV-2 by RT-PCR during this study and evaluated for cellular response are represented by triangles. The proportions of naive/memory T-cells that were SARS-CoV-2-specific CD4^+^ (CD137^+^OX40^+^) **(E)** and CD8+ (CD69^+^CD137^+^) **(F)** T-cells according to the AIM. CM, central memory; TEMRA, terminally differentiated cell; EM, effector memory. The responses 2 and 6 months after vaccination in Exposed and Non-Exposed individuals were compared using a Wilcoxon paired test (*p<0.05 and **p<0.01).

Cellular responses to the SARS-CoV-2 peptide pool through an AIM assay (**Supplementary Figure S2**) revealed that most vaccinated children had detectable virus-specific AIM^+^CD4^+^ (coexpressing either CD137/OX40 or OX40/CD25), irrespective of the SARS-CoV-2 variant (WT or Omicron), SARS-CoV-2-exposure, or time post-vaccination (**Figures 3A, B**).

In contrast, the frequency of AIM^+^CD8^+^ T-cells (CD69^+^CD137^+^) in response to Omicron was lower than to WT in both Exposed (p=0.043) and Non-Exposed (p=0.011) at 6 months post-vaccination (**Figure 3C**). The CD25/CD107a combination also detected these differences at 2 months post-vaccination (p<0.05, **Figure 3D**). More children responded through AIM^+^CD4^+^ than AIM^+^CD8^+^ T-cells (p<0.05, Fisher’s test), regardless of the variant or time post-vaccination. Neither variants, exposure status, nor time post-vaccination affected the memory profile of virus-specific T-cells, with predominant central memory CD4^+^ T-cells and effector memory CD8^+^ T-cells (**Figures 3E, F**).

**Figure 4:**
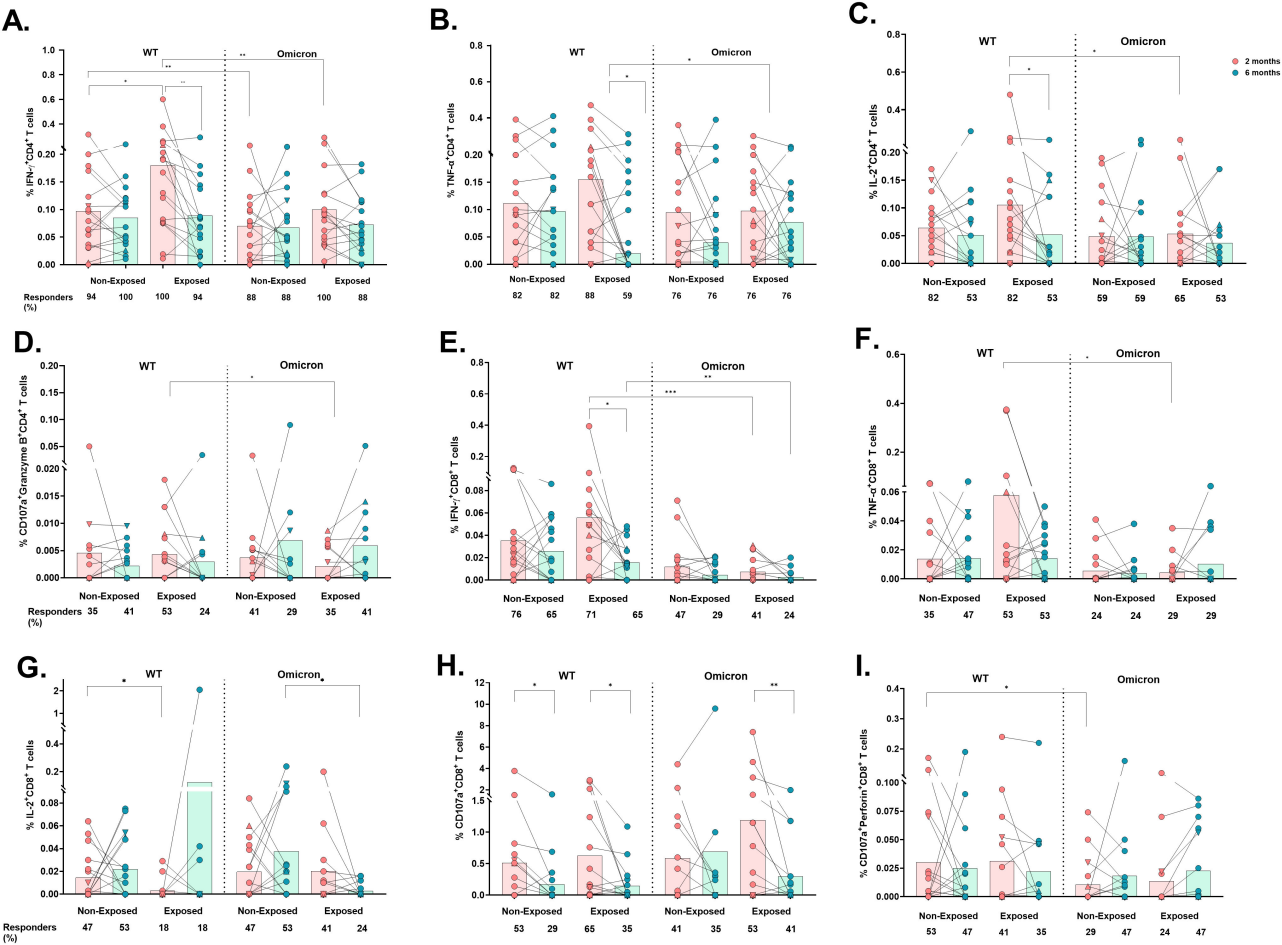
Cytokine- and cytotoxicity-mediated CD4^+^ and CD8^+^ T-cell responses to the WT strain and the Omicron variant: The frequencies of CD4^+^ T-cells positive for IFN-γ **(A)**, TNF-α **(B)**, IL-2 **(C)**, CD107a/Granzyme B coexpression **(D)**, CD8^+^ T-cells positive for IFN-γ **(E)**, TNF-α **(F)**, and IL-2 **(G)** and CD107a expression **(H)** and CD107a/perforin coexpression **(I)** are shown (n=17 per group). Each point represents the CD4^+^ and CD8^+^ T cell response of an individual to each peptide pool (5 µg/mL) from the WT strain and Omicron variant at 2- or 6-months post-vaccination. The data were reported after background subtraction (from the negative control), and individuals were considered responders with a minimum threshold of 0.001%. In the figures, four out of seven children who tested positive for SARS-CoV-2 by RT-PCR during this study and evaluated for cellular response are represented by triangles. The Wilcoxon test for matched paired samples and the Mann−Whitney test were used to analyze differences between factors (viral variant, exposure to SARS−CoV−2 before vaccination, and time post-vaccination) (*). The bars represent the means. *p < 0.05, **p < 0.01, and ***p<0.001.

Cytokine and cytotoxic molecule production were subsequently analyzed in CD4^+^ and CD8^+^ T-cells after peptide stimulation (**Supplementary Figure S3**). Lower frequencies of IFN-γ^+^CD4^+^ (p*=*0.0041), TNF-α^+^CD4^+^ (p*=*0.0380) and IL-2^+^CD4^+^ T-cells (p*=*0.0214) were observed in response to Omicron than WT in the Exposed children, at 2 months post-vaccination; however, this response decreased over time, only in response to WT (IFN-γ^+^CD4^+^; p=0.0017, TNF-α^+^CD4^+^; p*=*0.0239 and IL-2^+^CD4^+^; p*=*0.0289. **Figures 4A–C**).

Similarly, IFN-γ^+^CD4^+^ T-cell frequency was also lower in response to Omicron compared to WT in Non-Exposed subjects at 2 months post-vaccination (*p=*0.0066. **Figure 4A**). The response to WT in Non-Exposed was slightly lower than in Exposed at 2 months post-vaccination (p*=*0.0447. **Figure 4A**). The frequencies of IFN-γ-, TNF-α-, and IL-2-producing CD4^+^ T-cells were positively correlated with the frequency of AIM^+^CD4^+^ T-cells (CD137^+^OX40^+^) in both groups, with correlation coefficients ranging from 0.49 to 0.61 (*p*<0.05, **Supplementary Figure S4**). No significant differences were found in IL-10^+^, CD107a^+^, nor CD107a^+^Perforin^+^ CD4^+^ T cell frequencies among any of the conditions or groups evaluated (**Supplementary Figures S5A–C**). However, CD4^+^ T-cells coexpressing CD107a^+^Granzyme B^+^ were higher in response to Omicron peptides compared to WT peptides in the Exposed group at 6 months post-vaccination (p*=*0.0391, **Figure 4D**).

Similar to those of CD4+ T-cells, the frequencies of IFN-γ^+^CD8^+^ (p*=*0.0010) and TNF-α^+^CD8^+^ (p*=*0.0273) T-cells were significantly lower in response to Omicron than in response to WT in Exposed children at 2 months (**Figures 4E, F**), and also at 6 months post-vaccination for IFN-γ^+^CD8^+^ T-cells (p*=*0.0093). The IFN-γ^+^CD8^+^ response to WT in Exposed subjects decreased at 6 months compared to that at 2 months (p*=* 0.0245. **Figure 4E**).

In addition, a greater percentage of responders and frequency of IL-2^+^CD8^+^ T-cells were observed in the Non-Exposed compared to the Exposed group in response to WT at 2 months (*p=*0.0458) and to Omicron at 6 months (p=0.0232) (**Figure 4G**). No significant differences were found in IL-10^+^CD8^+^ T-cell frequencies (**Supplementary Figure S5D**).

A significant decrease in the frequency of total CD107^+^CD8^+^ T-cells was observed at 6 months compared to 2 months post-vaccination in response to WT peptides in both Non-Exposed (p*=*0.0137) and Exposed (p*=*0.0356) groups, and in response to Omicron, only in the Exposed group (p*=*0.0059) (**Figure 4H**). The frequency of CD107a^+^perforin^+^-expressing CD8^+^ T-cells was lower in response to Omicron compared to WT in Non-Exposed children at 2 months post-vaccination (p*=*0.0391) (**Figure 4I**).

### 3.5 CoronaVac^®^ induces a polyfunctional T-cells response to viral variants

**Figure 5:**
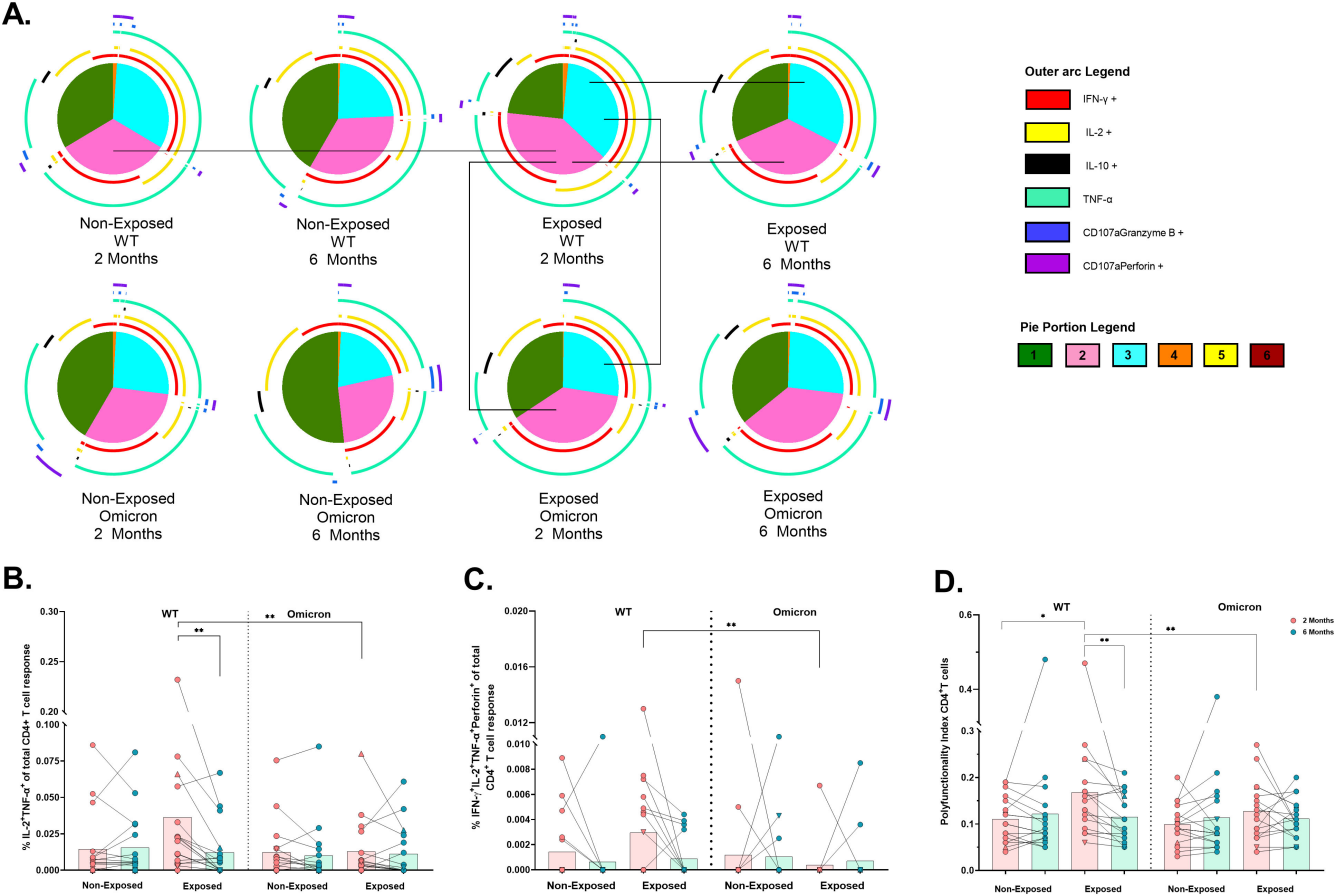
Functional profile of the SARS-CoV-2-specific CD4^+^ T cell response: Pie charts showing the proportions of combinations of IFN-γ, IL-2, IL-10, TNF-α, CD107a/granzyme B and CD107a/Perforin **(A)** of CD4^+^ T-cells in response to pooled peptides (5 µg/mL) from the WT strain and the Omicron variant at 2- or 6- months post-vaccination (n=17 per group). The size of the pie segment correlates to the frequency of the population. The arcs around the circumference indicate the molecule (see the legend) produced by the proportion of cells that lie under the arc. The parts of the pie surrounded by multiple arcs represent polyfunctional cells. Significant comparisons (p< 0.05) were marked with black lines between pie portions. A threshold equal to or higher to 0.001% was considered positive. The frequencies of CD4^+^ T-cells exclusively expressing IL-2^+^TNF-α^+^ **(B)** and IFN-γ^+^IL-2^+^TNF-α^+^perforin^+^ **(C)**, as well as the polyfunctionality index of CD4^+^ T-cells **(D)**, were analyzed with FunkyCell Toolbox software. Each point represents the CD4^+^ T cell response of an individual to each peptide pool (5 µg/mL) from the WT strain and the Omicron variant at 2- or 6-months post-vaccination. The data are reported after background subtraction (from the negative control). In the figures, four out of seven children who tested positive for SARS-CoV-2 by RT-PCR during this study and evaluated for cellular response are represented by triangles. The Wilcoxon test for matched paired samples and the Mann−Whitney test were performed according to the analysis. Bars depict the mean. *p< 0.05 and **p< 0.01.

Coronavac^®^ induced polyfunctional CD4^+^ T-cell responses, predominantly coexpressing TNF-α, IFN-γ, and IL-2, against both viral variants. However, the frequency of trifunctional (p*=*0.0012) and bifunctional (p=0.0215) cells was lower in response to Omicron compared to WT peptides in Exposed children at 2 months post-vaccination. The response to WT in Exposed children also decreased over time (3 functions: p*=*0.0203, 2 functions: p*=*0.0074). Similarly, the bifunctional response to WT was also lower in Non-Exposed compared to Exposed children at 2 months (p*=*0.0169) (**Figure 5A**).

Bifunctional IL-2^+^TNF^+^ cells in the Exposed children were lower in response to Omicron than WT at 2 months (p*=*0.0020) and this response to WT also decreased over time (p*=*0.0021) (**Figure 5B**). In addition, the IFNγ^+^IL-2^+^TNF-α^+^perforin^+^ profile was less frequent in response to Omicron than in response to WT in Exposed children at 2 months (p*=*0.0078) (**Figure 5C**). The polyfunctionality index in CD4^+^ T-cells was lower in response to Omicron than WT in Exposed children at 2 months. It was higher in Exposed than Non-Exposed children in response to the WT, decreasing over time (**Figure 5D**).

**Figure 6:**
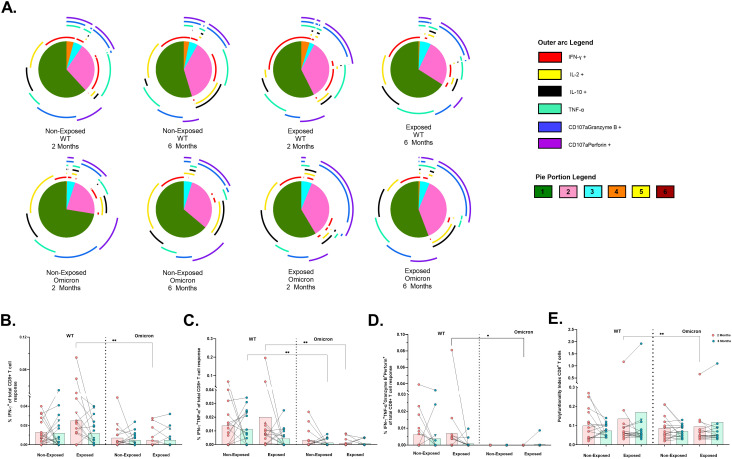
Functional profile of the SARS-CoV-2-specific CD8^+^ T cell response: Pie charts showing the proportions of combinations of IFN-γ, IL-2, IL-10, TNF-α, CD107aGranzyme B and CD107aPerforinB **(A)** of CD8^+^ T-cells in response to pooled peptides (5 µg/mL) from the WT strain and the Omicron variant at 2- or 6-months post-vaccination (n=17 per group). The size of the pie segment correlates to the frequency of the population. The arcs around the circumference indicate the molecule (see the outer arc legend) produced by the proportion of cells that lie under the arc. The parts of the pie surrounded by multiple arcs represent polyfunctional cells. A threshold equal to or higher to 0.001% was considered positive. The frequencies of CD8^+^ T-cells exclusively expressing IFN-γ^+^ **(B)**, IFN-γ^+^TNF-α^+^ **(C)**, IFN-γ^+^TNF-α^+^GranzymeB^+^Perforin^+^ **(D)**, as well as the polyfunctionality index of CD8^+^ T-cells **(E)**, were analyzed with FunkyCell Toolbox software. Each point represents the CD4^+^ T cell response of an individual to each peptide pool (5 µg/mL) from the WT strain and the Omicron variant at 2- or 6-months post-vaccination. The data are reported after background subtraction (*from the negative* control). In the figures, four out of seven children who tested positive for SARS-CoV-2 by RT-PCR during this study and evaluated for cellular response are represented by triangles. The Wilcoxon test for matched paired samples and the Mann−Whitney test were performed according to the analysis. Bars depict the mean. *p< 0.05 and **p< 0.01.

In contrast, CD8^+^ T-cells were less polyfunctional overall, with no significant differences across subjects (**Figure 6A**). Bifunctional responses against both variants were predominantly granzyme B^+^perforin^+^, followed by IFN-γ^+^ TNF-α^+^ profile for WT and IL-2^+^IL-10^+^ profile for Omicron (**Figure 6A**). A significant reduction in the frequencies of CD8^+^ T-cells exclusively expressing IFNγ^+^ (p*=*0.0032) (**Figure 6B**), IFNγ^+^TNF-α^+^ (p*=*0.0020) (**Figure 6C**) and IFN-γ^+^TNF-α^+^granzyme-B^+^perforin^+^ (*p =* 0.0156) (**Figure 6D**) was observed in response to Omicron compared to WT in Exposed children at 2 months. Additionally, the frequency of IFNγ^+^TNF-α^+^ profile was lower for Omicron than WT in Non-Exposed children at 6 months post-vaccination (p*=*0.0020) (**Figure 6C**). A higher polyfunctional CD8^+^ T-cell index was observed for WT compared to Omicron in Exposed children at 2 months post-vaccination (p*=*0.0097) (**Figure 6E**).

**Figure 7:**
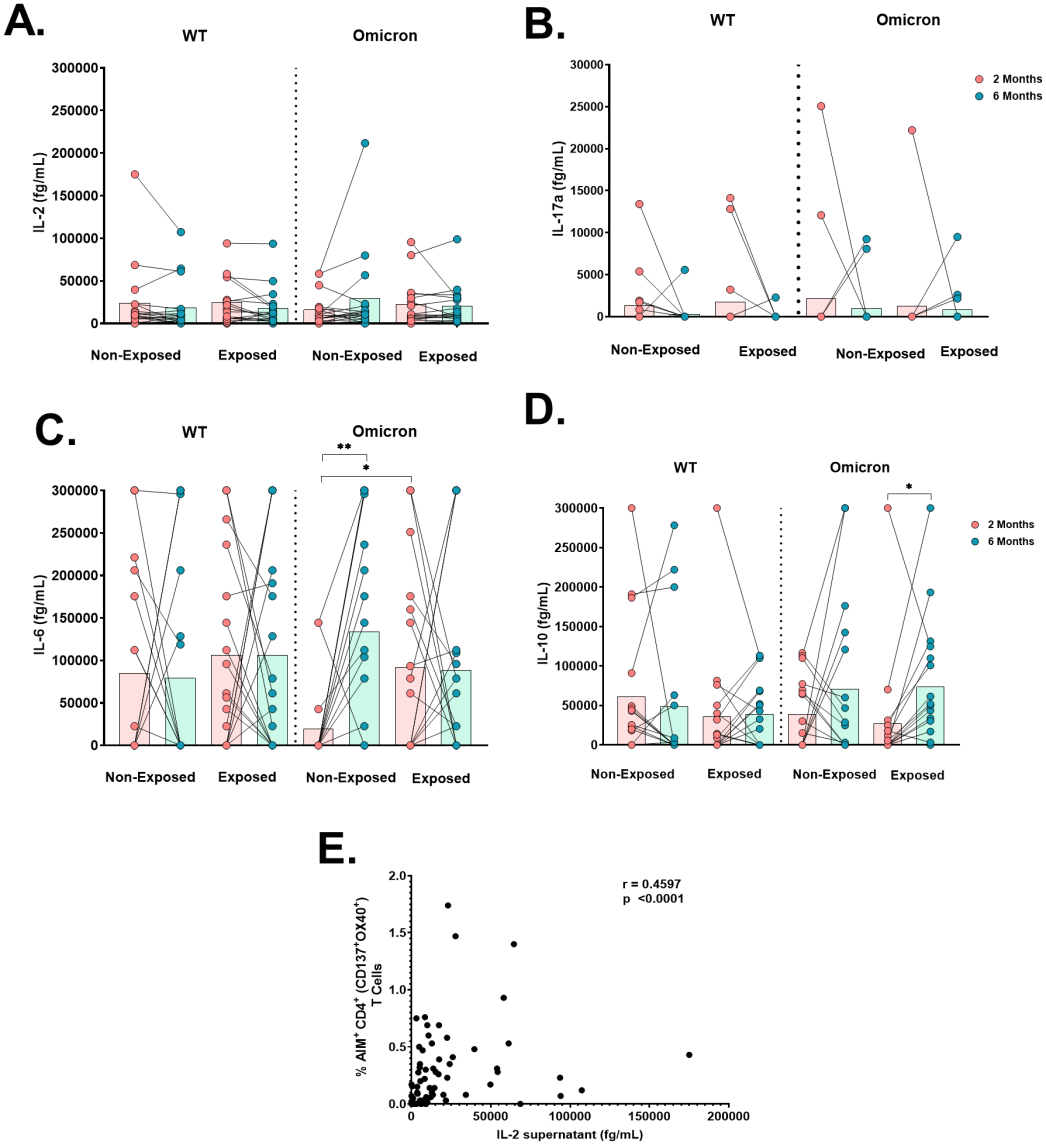
Cytokine production after antigen-specific stimulation: Antigen-specific cytokine production in the cell culture supernatant of peripheral blood mononuclear cells (PBMCs) at 2- or 6-months post-vaccination stimulated for 18 hours with SARS-CoV-2-specific peptides (5 µg/mL) from the WT strain and Omicron variant was evaluated through a cytometric bead assay (CBA) (n=17 per group). The data are reported after background subtraction (from the DMSO vehicle control). Concentrations expressed in femtograms for IL-6 **(A)**, IL-10 **(B)**, IL-2 **(C)**, and IL-17a **(D)** and the correlation between the levels of IL-2 produced in response to the WT strain in cell culture supernatants assessed by CBA and the frequency of AIM^+^CD4^+^ (CD137^+^OX40^+^) in Exposed and Non-Exposed subjects analyzed together **(E)** are shown. Correlations were evaluated by Spearman correlation Rank (r). *p< 0.05 and **p< 0.01 according to the Wilcoxon test for matched paired samples and the Mann−Whitney test. Bars depict the mean.

When cytokine production was measured in the supernatants of cultures stimulated with WT or Omicron peptides, IL-2 production was detected in all subjects (**Figure 7A**); IL-17A levels were low in all subjects (**Figure 7B**), and IL-4 was not detected. IL-6 levels in response to Omicron in Non-Exposed subjects increased at 6 months compared to 2 months post-vaccination (p=0.0027). Still, they were lower in Non-Exposed compared to Exposed at 2 months post-vaccination (p=0.019) (**Figure 7C**). IL-10 levels in response to Omicron in the Exposed group increased at 6 months compared to 2 months post-vaccination (p=0.0107) (**Figure 7D**). The IL-2 levels in response to WT were positively correlated with the frequency of AIM^+^CD4^+^ (CD137^+^OX40^+^; r=0.4597, p<0.0001) T-cells in Exposed and Non-Exposed subjects analyzed together (**Figure 7E**). However, these correlations were not observed for IL-2 produced in response to Omicron.

## 4 Discussion

To our knowledge, this is the first real-world study on CoronaVac^®^ immunogenicity in Latin American children. Our findings align with previous studies demonstrating that this vaccine is well-tolerated and safe (8). Consistent with earlier evidence showing significant IgG antibody production induced by CoronaVac^®^ in children (9) and adults (10, 11), our results also provide new evidence of IgA induction in children. Whereas previous studies have shown that CoronaVac^®^ (12) or natural infection (13) do not induce IgA in adults, others have reported increased serum IgA, but not mucosal IgA, in comparison to mRNA vaccines in adults (14) and children (15). This variability suggest that although germinal center responses likely occur following CoronaVac^®^ vaccination, further research should focus on redirecting memory B cells towards mucosal tissues. Studying SARS-CoV-2-specific IgA in nasal mucosa and saliva, as has been done for mRNA vaccines (16), could help elucidate the IgA role in CoronaVac^®^-induced protection in children. As expected, IgG and IgA levels declined six months post-vaccination, consistent with previous studies on CoronaVac^®^ in adults (17, 18) and children, at least for IgG (10).

Notably, CoronaVac^®^ induced robust neutralizing activity against the ancestral lineage (B.1) and the Delta, Mu, and Omicron (BA.1.1) variants, regardless of prior exposure or time. Although these titers decreased over time, most participants maintained neutralizing titers ≥20, as previously shown (6). These findings align with those of efficacy studies showing that despite a waning humoral response, CoronaVac^®^ protects against severe disease and death by 90.3% and 80.6%, respectively (19). The observed decline in neutralization over time against Omicron in both Exposed and Non-Exposed children, consistent with other studies (20), underscores the potential benefit of booster doses to enhance neutralization against variants.

Interestingly, we found higher neutralizing titers against Delta and Mu than against B.1 at two- and six-months post-vaccination, which could be attributed to original antigenic sin (OAS), also known as antigenic seniority, in which the immune response to a primary antigen dominated over newer strains in subsequent exposures (21, 22). Thus, the higher titers against Delta and Mu than B.1 likely reflect the circulation of these variants in Colombia before vaccination (23). The OAS phenomenon has been previously reported against SARS-CoV-2 (24) and other respiratory viruses such as influenza (25). Epidemiological data suggest that the Delta wave in Colombia had reduced severity compared to other countries, potentially due a robust immune response against Delta because of prior Mu infection, which has less antigenic distance from Delta than B.1 and other variants. In contrast, primary exposures to B.1 or other variants with greater antigenic distance from Delta resulted in more severe Delta infections elsewhere (21, 26). Moreover, the evidence of reduced neutralizing efficacy of CoronaVac^®^ against SARS-CoV-2 variants, including Delta, Mu, and Omicron, due to mutations in key RBD regions (27–29), has been found in individuals exposed for the first time to the Wuhan strain (22).

CoronaVac^®^ elicited specific and functional CD4^+^ and CD8^+^ T-cell responses against WT and Omicron, as shown by AIM combinations and ICS assays. However, the response of monofunctional cells (IFN-γ, IL-2 or TNF-α) and cells with two (IL-2^+^TNF-α^+^) or three functions (IFN-γ^+^IL-2^+^TNF-α^+^) to Omicron was lower than that to WT in the Exposed group at 2 months post-vaccination. Responses against WT, particularly in Exposed children, decreased from 2 to 6 months probably reflecting a natural contraction of the immune response. The lower response to Omicron, also observed in other studies, may be due to mutations in immunodominant epitopes affecting T-cell activation (30). Hybrid immunity resulting from prior infection and vaccination may explain differences in responses to WT versus Omicron and over time, particularly in Exposed individuals (31).

Notably, most children who tested positive for SARS-CoV-2 by RT-PCR during this study (represented by triangles in the figures) consistently exhibited several immune parameters evaluated above the average, reinforcing the idea that hybrid immunity enhances immune responses. Indeed, persisting SARS‐CoV‐2 antibodies and robust CD4 and CD8 T‐cell activation and polyfunctional responses have been observed in vaccinated children following Omicron infection (32). However, the minimal differences observed between Exposed and Non-Exposed children could be attributed to undocumented SARS-CoV-2 exposures or asymptomatic infections in Non-Exposed children before vaccination. In fact, SARS-CoV-2-infected individuals and their uninfected close contacts have exhibited virus-specific CD4^+^ and CD8^+^ T-cell memory, with larger and higher-quality T-cell responses in infected individuals (33). Furthermore, both asymptomatic and symptomatic infected patients have shown similar levels of antibodies (34, 35) and virus-specific T-cell memory (33). Thus, Non-Exposed may have experienced undetected SARS-CoV-2 exposures or asymptomatic infections, which could have modulated the immune parameters evaluated (33). Consistent with high asymptomatic infection rates in children, only two of seven children infected during this study presented mild symptoms (35).

CoronaVac^®^ predominantly triggered polyfunctional CD4^+^ T-cells coexpressing TNF-α, IFN-γ, and IL-2 but at lower levels against Omicron than against WT. Overall, antigen-specific CD4^+^ T cell responses decreased by 6 months, stabilizing at similar levels in both groups, indicating physiological contraction of the immune response. These cells exhibited effector and central memory phenotypes, similar to natural infection and after vaccination (36), potentially sustained by IL-2 and TNF-α, crucial for memory T-cell proliferation and survival (37, 38). CoronaVac^®^ predominantly triggered a Th1 response associated with viral control (39), low IL-17, and no IL-4, consistent with earlier findings (4). However, Omicron increased IL-6 and IL-10 secretion over time, suggesting a Th2-like profile that might interfere with the antiviral response (40). Additionally, a cytotoxic response by CD4^+^ T-cells (CD107a^+^ granzyme B^+^) against Omicron was observed in the Exposed group at the end of the follow-up, possibly stimulated by IL-2 (41). At two months post-vaccination, a secretory and cytotoxic response of CD4^+^ T-cells (IFN-γ^+^IL-2^+^TNF-a^+^perforin^+^) against WT was noted in the Exposed group, suggesting a hybrid immunity with a broader antiviral response. Although prior studies suggested CD4^+^ T-cell cytotoxic responses (IFN-γ^+^ CD107a^+^) post-mRNA vaccination, further research is needed to elucidate this aspect (42).

In contrast to CD4^+^, CD8^+^ responses were more variable and often lower, mostly monofunctional. This CD4/CD8 imbalance, seen in response to natural infection and vaccination (4, 43), might be due to the inactivated nature of CoronaVac^®^ and the aluminum hydroxide adjuvant favoring CD4^+^ over CD8^+^ T-cell (44). CD8^+^ T responses, including IFN-γ^+^, TNF-α^+^, bifunctional cells (IFN-γ^+^TNF-α^+^ and CD107a^+^IFN-γ^+^), and the polyfunctionality index were reduced in response to Omicron, consistent with AIM assays. AIM^+^CD8^+^ T-cells were primarily TEM and TEMRA cells, resembling those induced by post-natural infection and other vaccines (45), indicating robust cytolytic responses despite a limited CD8^+^ T-cell pool. The greater decline in CD8^+^ compared to CD4^+^ T-cell responses, along with the weaker response to Omicron, reinforces the need for booster doses or adjuvants favoring cross-presentation and CD8^+^ T-cells, such as Alum-based nanoparticles, and Matrix-M that could help to prime a robust response of CD8^+^ T cells (44).

The strong humoral and cellular responses to SARS-CoV-2 elicited by CoronaVac^®^ in children align with evidence showing comparable or superior humoral responses in children relative to adults in terms of serologic response rates, duration, and both antibody and neutralizing titers (46, 47). Additionally, children have been observed to sustain longer-lasting neutralizing and binding antibody responses (48) and exhibit a higher proportion of IFN-γ-producing T cells after SARS-CoV-2 infection than adults (49). Despite reduced cellular responses to Omicron, they were still detectable. Moreover, the robust neutralizing antibodies may compensate, providing adequate protection against severe COVID-19 in pediatric populations during the Omicron surge when this variant predominated during recruitment (50, 51).

A key limitation of this study was the inability to accurately assess the Non-exposed group due to the possibility of undocumented SARS-CoV-2 exposures or asymptomatic infections before the study’s initiation. The absence of pre-vaccination samples prevented establishing a true baseline, potentially leading to false Non-Exposed cases. Despite these challenges, recognized from the outset, they are common in real-world research, especially given estimates of widespread SARS-CoV-2 exposure in approximately 90% of the population since the pandemic began.

In conclusion, CoronaVac^®^ elicits robust humoral and cellular immune responses, including total and neutralizing antibodies, as well as SARS-CoV-2-specific Th1-like CD4^+^ and CD8^+^ polyfunctional T-cell responses, characterized by IFN-γ production and cytotoxic capacity. Nevertheless, waning immunity and reduced responses to variants such as Omicron underscore the importance of appropriately timed, heterologous booster doses with optimized adjuvants and variant-specific antigens to enhance the magnitude, breadth, and durability of the immune response (52), particularly against emerging strains such as JN.1 and KP.2. Continuous genomic surveillance is essential for guiding vaccine updates, particularly in low- and middle-income countries, where vaccine performance may differ from trial results in high-income settings. Addressing these gaps will improve global preparedness for current and future SARS-CoV-2 outbreaks.

## Data Availability

The raw data supporting the conclusions of this article will be made available by the authors, without undue reservation.
