# Supplementary material for: Immunogenicity of the CoronaVac vaccine in children: a real-world study
Source: Front Immunol. 2024 Dec 23;15:1504935. doi: 10.3389/fimmu.2024.1504935 (PMC11700979; doi:10.3389/fimmu.2024.1504935)
Supplement: Supplementary file 1 [file DataSheet1.pdf]

Supplementary material

"Immunogenicity of the CoronaVac Vaccine in Children: A Real-World Study"

Supplementary Table S1: Reagents

**Uses**	**Product name**	**Clone**	**Catalogue number**	**Company**
**Surfaces antibody (AIM Assay)**	Fixable Viability Dye eFluor506		304140	Biolegend
	anti-CD3-Alexa fluor 700	UCHT1	56-0038-42	Thermo
	anti-CD4-APC-eFluor 780	OKT-T4	47-0049-42	Thermo
	anti-CD8-Pe-eFluor610	RPA T8	61-0088-42	Thermo
	anti-CD25-Pe-Cy5	M-A251	555453	Beckton Dickinson
	anti-134 (OX40)-PE-Cyanine7	ACT35	350012	Biolegend
	anti-CD69- PerCP-eFluor710	FN50	46-0699-92	Invitrogen
	anti-CD137 (4-1BB)-PE	4B4-1	309804	Biolegend
	anti-CD45RA-Brilliant violet 785	H100	304140	Biolegend
	anti-CD197 (CCR7)-FITC	150503	561271	Beckton Dickinson
	Anti-CD107a-APC	H4A3	641581	Beckton Dickinson
**Intracellular antibody (ICS)**	anti-IFN-γ-PE Cy7	4SB3	25-7319-82	Thermo
	anti-TNF-α- PerCP. Cy5.5	Mab11	45-7379-42	Invitrogen
	anti-IL-2-BV605	534411	563947	Beckton Dickinson
	anti-IL-10-eFluor450	JES3-9D7	48-7108-42	Thermo
	anti-perforin-PE	B-D48	353304	Biolegend
	anti-Granzyme B-FITC	GB11	560211	Beckton Dickinson
**Stimulus**	Anti-CD28	CD28.2	16-0289-85	Thermo
	Anti-CD49d	9F10	16-0499-85	Thermo
**Cytokines detection by cytometry beads assay**	Human IL-2 Enhanced Sensitivity Flex Set		561517	Beckton Dickinson
	Human IL-4 Enhanced Sensitivity Flex Set		561510	Beckton Dickinson
	Human IL-6 Enhanced Sensitivity Flex Set		561512	Beckton Dickinson
	Human IL-10 Enhanced Sensitivity Flex Set		561514	Beckton Dickinson
	Human IL-17A Enhanced Sensitivity Flex Set		562143	Beckton Dickinson
	BD CBA Human Enhanced Sensitivity Master Buffer Kit		561523	Beckton Dickinson

Supplementary Table S1: Reagents. Cont….

**Uses**	**Product name**	**Catalogue number**	**Company**
**Molecular Biology**	DNA/RNA Shield (2X Concentrate)	R1200-125	Zymo
	SaMag Viral Nucleic Acids Extraction kit	SM003	Sacace Biotechnologies
	Real-Time Fluorescent RT-PCR Kit for Detecting SARS-2019-nCoV	MFG030010	BGI Genomics
**Antibody quantification**	COVID 19 Spike S IgG Coronavirus (COVID-19), ELISA Kit	MBS7612298	MyBioSource
	COVID 19 IgA Coronavirus (COVID-19), ELISA Kit	MBS7612290	MyBioSource
**Neutralization assays**	Trypsin-EDTA Solution 0.25%	T4049-500ML	Sigma
	carboxymethyl cellulose sodium salt	C4888-500G	Sigma
	Violeta cristal (C.I. 42555)	101408	Merk
	Formaldehyde solution 37%	818708	Sigma
	Foxp3/Transcription Factor Staining Buffer Set	00-5523-00	Thermo
	Phosphate-buffered saline (PBS)	D8537	Sigma
	Fetal Bovine Serum (FBS)	12106C	Sigma
	RPMI-1640 Medium	R8758	Sigma
	Dimethyl sulfoxide	102952	Merck
**Cell culture**	HISTOPAQUE-1077	10771	Sigma
	Phytohemagglutinin PHA-P, lyophilized Powder (PHA-P)	L8754	Sigma
	Phorbol 12-myristate 13-acetate	P8139-1MG	Sigma
	Ionomycin calcium salt from Streptomyces conglobatus powder	I0634-1GR	Sigma
	Brefeldin A	00-4506-51	Thermo
	Monensin	00-4505-51	Thermo
**Plastic Material**	96-well Clear V-Bottom TC-treated Microplate	3896	Corning

Supplementary Table S2: Peptides database

This database includes information of peptides, viral proteins and HLAs, due to its size, it is attached independently as a spreadsheet.

Supplementary figure S1: Representative plaques of neutralization assay

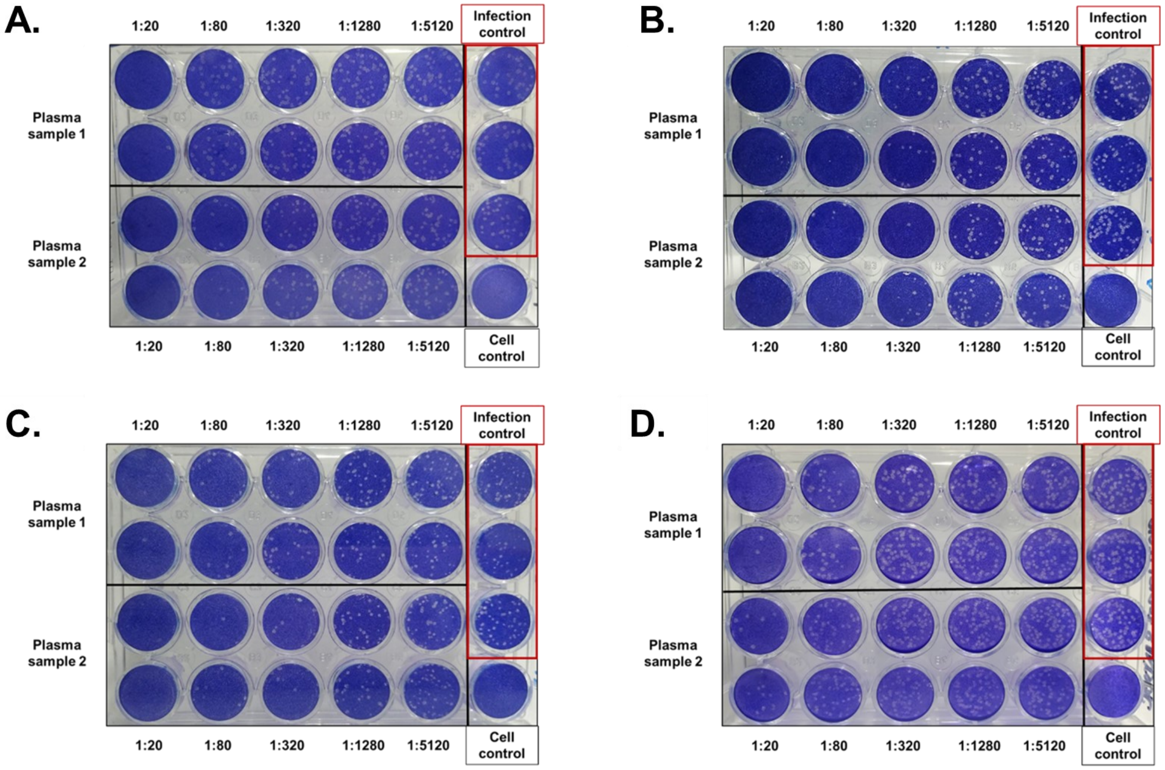

The figure shows representative plaques obtained from plaque reduction neutralization test (PRNT) for B.1 lineage (A) and, Delta (B), Mu (C), and Omicron (D) variants in Vero E6 cells. Five serial dilutions of each sample (from 1:20 to 1:5120) were done. For assays, infection controls (infected cells without plasma) and cell controls (cells without infection and without plasma) were included.

Supplementary figure S2. Flow-cytometry Gating strategy for detection of AIM$^+$CD4$^+$ and AIM$^+$CD8$^+$ T cells.

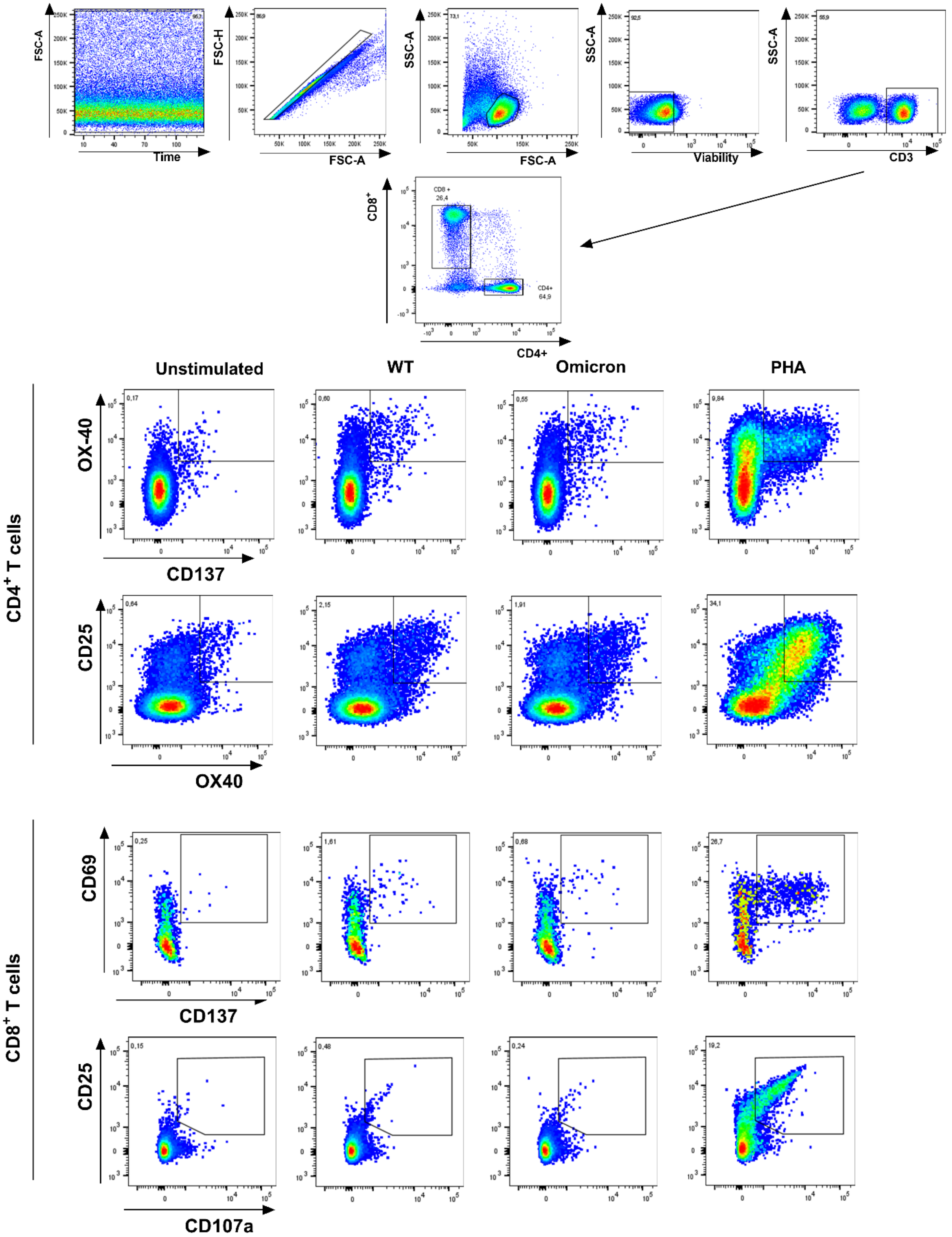

Exemplary plots showing the percentage of AIM$^+$ CD4$^+$ T cells (CD25$^+$OX40$^+$, CD137$^+$OX40$^+$) and AIM$^+$ CD8$^+$ T cells CD69+CD137+ CD25$^+$CD107a$^+$) virus-specific cells within gated CD4$^+$ T cells.

Supplementary figure S3. Flow-cytometry Gating strategy for detection of cytokines and cytotoxic molecules in CD4+ and CD8+ T cells.

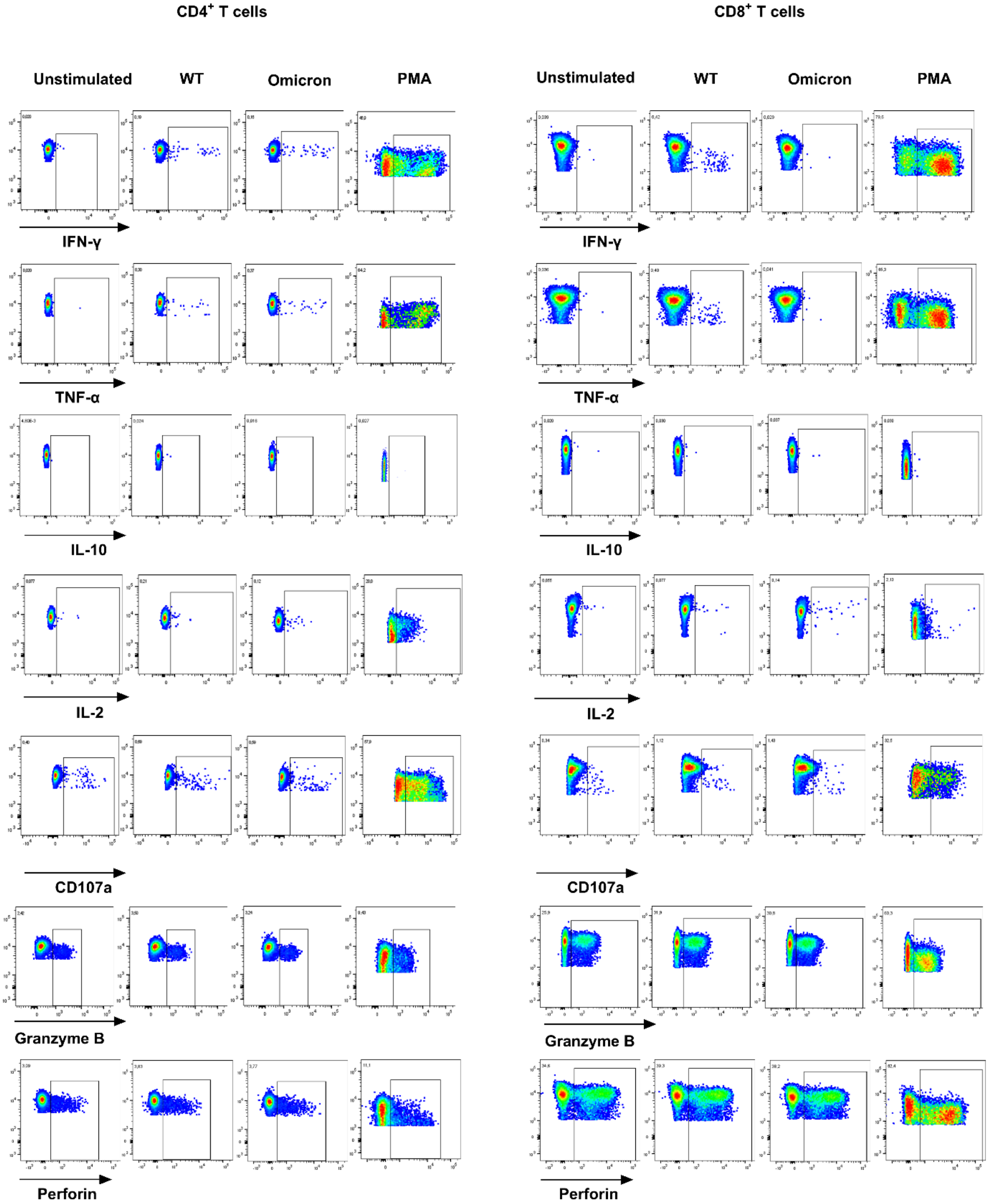

Gating strategy of CD4+ T cells and CD8+ T cells to identify the IFN-ɣ, IL-2, IL-10, TNF-a production, and the expression of CD107a, granzyme B and perforin by flow cytometry. As shown in Supplementary figures 18 and 20, a time gate was used to remove background noise and the cellular aggregates using a plot FSC-H Vs. FSC- Next, we selected the lymphocyte region and excluded dead cells using a viability stain. Each marker was analyzed from CD4+ or CD8+ T cells expressing CD3. Numbers inside the gates indicate frequencies within T cells.

Supplementary figure S4. Correlations between AIM^{+}CD4^{+} and cytokine-expressing CD4^{+} T cells.

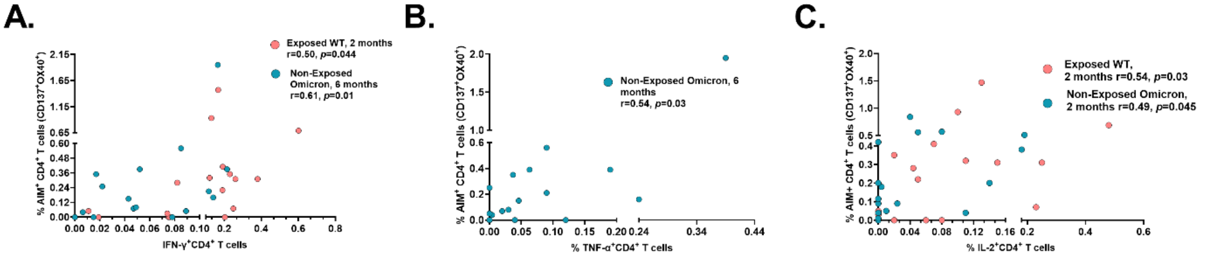

The frequency of AIM+CD4+ (CD137^{+}OX40^{+}) was positively correlated with the frequency of IFN-γ- (A), TNF-α- (B), and IL-2-producing (C) CD4^{+} T cells. Correlations were made by Spearman correlation Rank (r) in Exposed (blue circles) or Non-Exposed subjects (red circles).

Supplementary figure S5. Cytokine and cytotoxic mediated CD4+ and CD8+ T cell response to WT strain and Omicron variant.

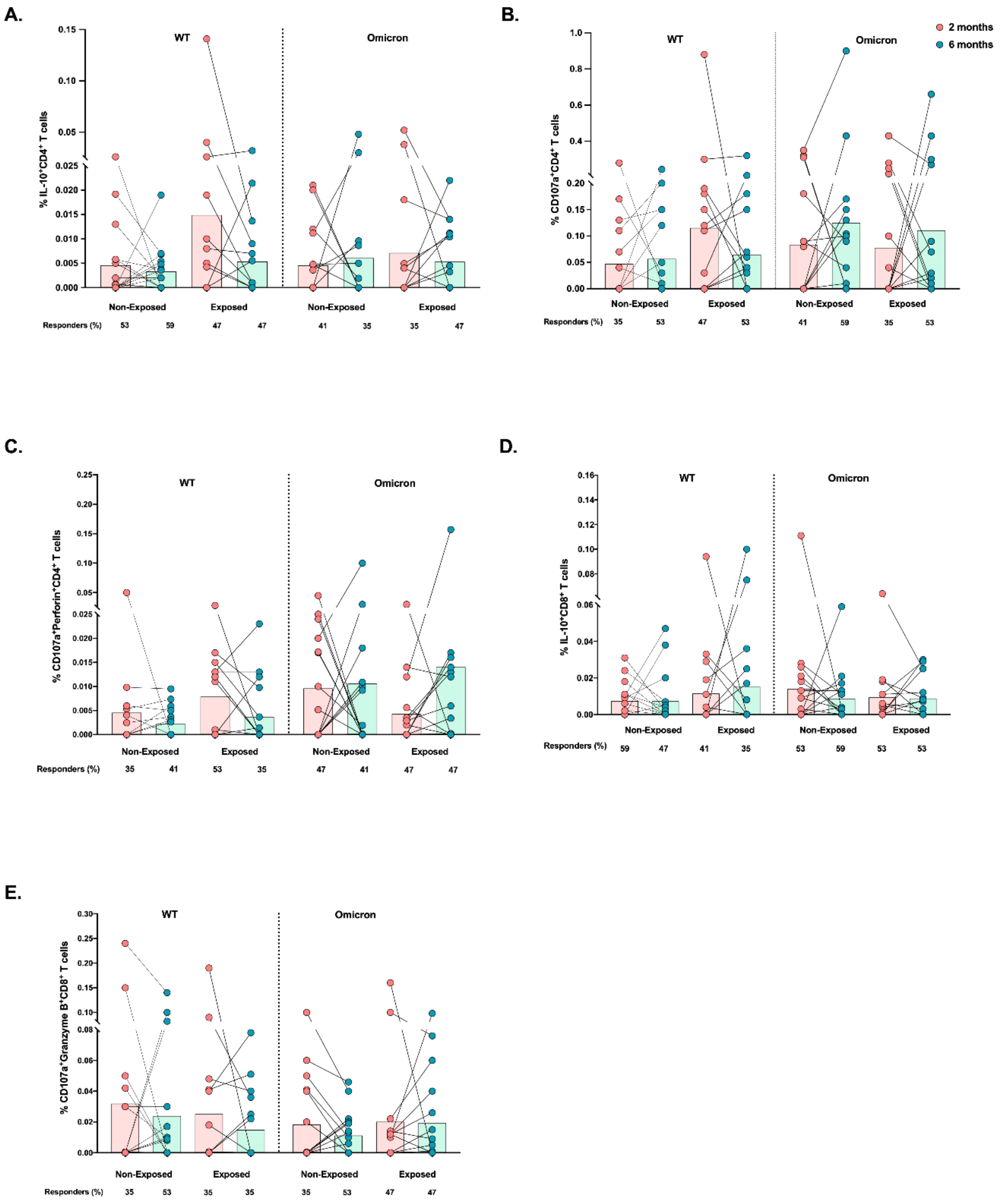

Frequency of CD4+ T cells positive for IL-10 (A), CD107a (B), CD107 and perforin (C), frequency of CD8+ T cells positive for IL-10 (D), and co-expressed CD107a and granzyme B (E). Each point represents the CD4+ T cell response in an individual to each peptide pool (5 µg/mL) from WT strain and Omicron variant after 2- or 6-months post-vaccination. Data were reported after background subtraction (from the negative control), and the individuals were considered responders with a minimum threshold of 0.001%. Wilcoxon test for matched-paired samples and Mann-Whitney test were done to analyze the differences between factors (viral variant, exposure to SARS-CoV-2 before vaccine, and time post-vaccination).
